# Zero-Effort Ambient Heart Rate Monitoring Using Ballistocardiography Detected Through a Seat Cushion: Prototype Development and Preliminary Study

**DOI:** 10.2196/25996

**Published:** 2021-05-31

**Authors:** Ahmed Raza Malik, Jennifer Boger

**Affiliations:** 1 Department of Systems Design Engineering University of Waterloo Waterloo, ON Canada; 2 Research Institute for Aging Waterloo, ON Canada

**Keywords:** ballistocardiography, heart rate, ambient health monitoring, zero-effort technology, continuous wavelet transform

## Abstract

**Background:**

Cardiovascular diseases are a leading cause of death worldwide and result in significant economic costs to health care systems. The prevalence of cardiovascular conditions that require monitoring is expected to increase as the average age of the global population continues to rise. Although an accurate cardiac assessment can be performed at medical centers, frequent visits for assessment are not feasible for most people, especially those with limited mobility. Monitoring of vital signs at home is becoming an increasingly desirable, accessible, and practical alternative. As wearable devices are not the ideal solution for everyone, it is necessary to develop parallel and complementary approaches.

**Objective:**

This research aims to develop a zero-effort, unobtrusive, cost-effective, and portable option for home-based ambient heart rate monitoring.

**Methods:**

The prototype seat cushion uses load cells to acquire a user’s ballistocardiogram (BCG). The analog signal from the load cells is amplified and filtered by a signal-conditioning circuit before being digitally recorded. A pilot study with 20 participants was conducted to analyze the prototype’s ability to capture the BCG during five real-world tasks: sitting still, watching a video on a computer screen, reading, using a computer, and having a conversation. A novel algorithm based on the continuous wavelet transform was developed to extract the heart rate by detecting the largest amplitude values (J-peaks) in the BCG signal.

**Results:**

The pilot study data showed that the BCG signals from all five tasks had sufficiently large portions to extract heart rate. The continuous wavelet transform–based algorithm for J-peak detection demonstrated an overall accuracy of 91.4% compared with electrocardiography. Excluding three outliers that had significantly noisy BCG data, the algorithm achieved 94.6% accuracy, which was aligned with that of wearable devices.

**Conclusions:**

This study suggests that BCG acquired through a seat cushion is a viable alternative to wearable technologies. The prototype seat cushion presented in this study is an example of a relatively accessible, affordable, portable, and unobtrusive zero-effort approach to achieve frequent home-based ambient heart rate monitoring.

## Introduction

Cardiovascular diseases are a leading chronic illness and are cited as the cause of death for nearly 17.9 million people worldwide every year [1]. In Canada alone, 2.4 million people are living with a diagnosed heart condition [2]. Cardiovascular diseases have large associated costs, which are estimated to exceed US $1 trillion by 2035 [3]. Population aging is one of the most significant social changes of the century, in part because the number of older adults with chronic health conditions who are living independently is increasing [4]. Most chronic conditions require frequent and continuous monitoring of vital signs and other health information to support ongoing treatment. The rapidly increasing number of older adults, who have a higher prevalence of chronic conditions, is leading to an unavoidable and significant increase in health monitoring for our global population [4,5].

The most important step toward the prediction, prevention, and treatment of cardiovascular diseases is cardiac vital monitoring, as it provides important information about a person’s cardiac health, which, in turn, supports ongoing management and care [6,7]. This is performed through routine visits to a clinic to record vital signs (ie, measures of the state of one’s body, including heart rate, blood pressure, temperature, and respiration). In addition to the financial cost and resources required to conduct a clinical assessment, accessing a clinical setting on a regular basis is not feasible for many people. This is especially true for people with limited mobility, who live in rural or remote areas or who have cognitive decline (eg, dementia). These situations can make frequent trips to a clinic for vital sign measurements expensive, difficult, and unrealistic.

To address this need, there is an increasing demand for technologies that enable the monitoring of vital signs from one’s home. The most common method for at-home vital sign monitoring is wearables [8-13]. Wearables are smart electronic devices that can be worn as accessories or integrated into clothing, such as smartwatches or smart clothing. Although wearables can be effective, they are not an ideal or feasible solution for everyone. Incorrect usage, noncompliance, and instances where users forget to use them can cause these technologies to be ineffective. These considerations are especially relevant for older adults, as they tend to have a lower adoption rate of monitoring technologies and have more difficulties using them. Older adults also have a much higher prevalence of cognitive impairment, such as dementia, which can make it difficult or impossible to intentionally and reliably interact with, wear, or charge a technology.

Ambient assisted living (AAL) is increasingly being used to support independent living, namely, information and communication technologies that support healthy living and well-being. AAL systems can monitor a person’s health status using sensors installed in their environment (eg, their home). Zero-effort technologies (ZETs) are a special class of technologies relevant to this area that are designed to require minimal or no explicit effort from the person using them. In this way, ZETs support users in such a way that they do not need to make modifications to their daily life activities nor do they need to focus their attention on the ZET to get support from it [14]. There has been some development in textile-based clothing for vital monitoring using textile electrodes, conductive fibers, and optical sensors [8-13]. However, these systems are not yet feasible because of issues related to cost, comfort, and durability. Therefore, given the increased costs and decreased feasibility of clinical monitoring and the problems associated with technologies such as wearables, there is room for improvement in at-home cardiac monitoring with easy-to-use technologies that operate autonomously.

While clinical monitoring and wearable technologies use electrocardiography (ECG) and photoplethysmography (PPG), another method of obtaining cardiac vital signs is through ballistocardiography (BCG). BCG is a cardiovascular signal that corresponds to the measurement of recoil forces generated by the body in response to blood flowing through a person’s vascular system [15,16]. Every time the heart beats, blood is pumped throughout the body, leading to a change in the center of mass. Microforces are then generated in the body as a response to the heart pumping blood to maintain the overall momentum. BCG is a recording of these micromovements and can be obtained using appropriate transducers, such as displacement, force, or acceleration.

BCG was first observed in 1877 [17], but ECG became the fundamental cardiovascular signal for clinical assessment because the noisy nature and hardware requirements of BCG were not practical during most of the last century. Since the 1990s, the scientific community has revisited BCG because of its simpler and more compliant instrumentation hardware and modern signal processing methods. This has resulted in the development of many BCG-based systems for cardiac monitoring and assessment, which are discussed as follows.

BCG has commonly been acquired in a standing position using a platform incorporated with force sensors, such as a bathroom scale [17], force plate [18], or custom-built floor tiles [19]. Although the standing upright position provides the least distorted BCG signals [20], a disadvantage of this approach is that the noise caused by the person moving to maintain balance is far greater than the BCG signal itself; therefore, the balance-induced noise masks the BCG signal. The measurement duration is often limited, as a person generally only stands still for only a few seconds at a time, even in specific locations such as in front of a sink. Wearable BCG systems have been reported in the literature. These systems use low-noise accelerometers to obtain BCG [21,22]. Wearable BCG systems are prone to the same noise issues as standing-position BCG systems as well as compliance and maintenance issues related to wearables, in general.

There has been some progress in BCG acquisition methods in the seated position. Most of these systems use electromechanical film sensors to obtain BCG, which is a charged polypropylene film that undergoes changes in the charge when pressure is applied to its surface [23]. Most chair-based systems have sensors embedded in the back or seat of a chair [24-27]. A toilet seat–based cardiovascular monitoring system has been reported to obtain BCG, ECG, and PPG from sensors embedded within the seat [28]. The seated position mitigates much of the noise interference problems associated with the standing position, as people tend to remain still in a seated position for a long period. However, current BCG systems in the seated position have disadvantages; most of these systems have used films, which are costly and have very limited commercial availability, and these systems are usually installed in a piece of furniture, which is less practical to do and not portable (ie, you need a special chair and it cannot be moved easily).

There is a need for novel solutions for cardiac monitoring that are autonomous, portable, and cost-effective. This research focuses on the development of an unobtrusive, portable, zero-effort seat cushion that uses BCG for cardiac monitoring.

## Methods

### Overview

To develop a method for BCG acquisition that is portable, easy to integrate into most environments, and requires minimal effort from the user, a seat cushion was chosen as the form factor of the proposed prototype. As BCG corresponds to recoil forces in the body due to blood flow, load cells are commonly used to sense and convert these forces to electrical signals and are a robust, well-understood sensor. To ensure minimal cost and relative ease of development for the prototype, a commercial weighing scale (with load cells installed underneath) was modified and inserted into the seat cushion, as described in the following section.

### Seat Cushion Prototype

**Figure 1:**
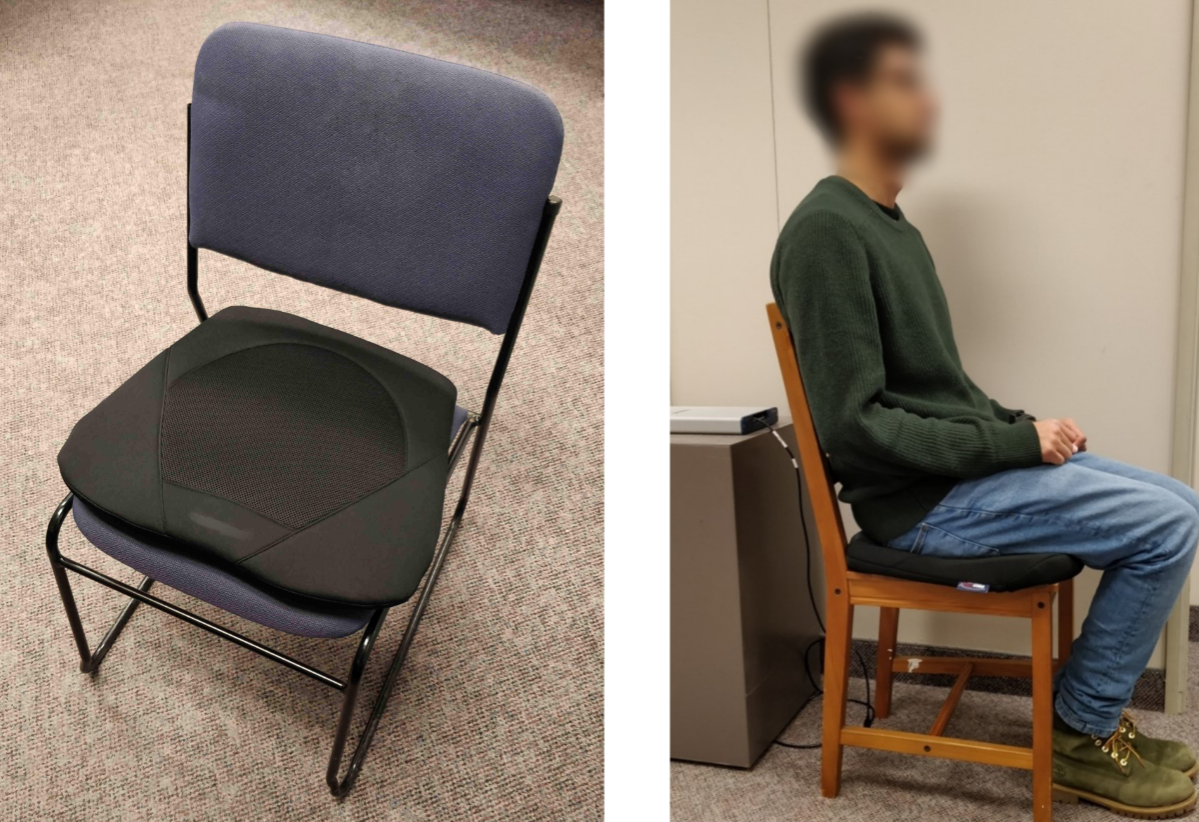
The developed prototype seat cushion (left) and a participant seated on the cushion (right).

Figure 1 shows the seat cushion prototype. The seat cushion was constructed by modifying an ObusForme Gel Seat cushion and consists of three layers. The top layer is a polyurethane foam wrapped over and around a modified weighing scale (NY-H05), which forms the second layer. The weighing scale has four strain gauge–type load cells, one mounted on each corner of the bottom of the scale. The third and bottom layer is a custom-built thin (0.8 mm) metal plate placed under the modified weighing scale, so that the load cells were placed on a solid surface.

### Signal Conditioning and Data Acquisition

**Figure 2:**
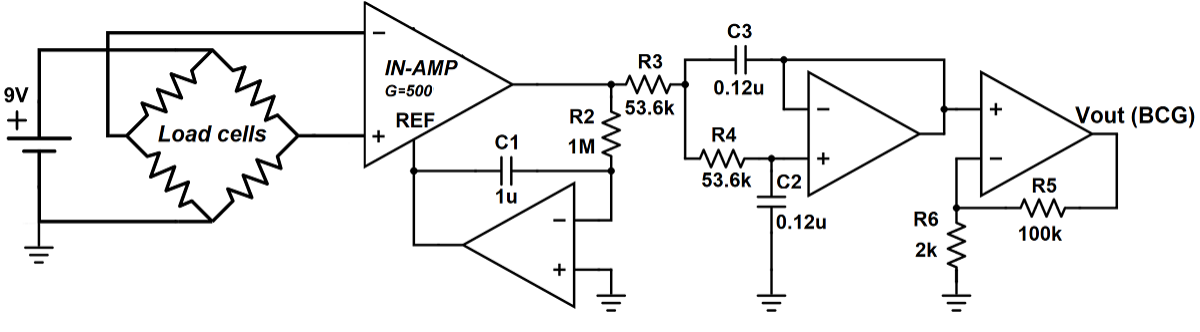
The analog signal-conditioning circuit. BCG: ballistocardiography; IN-AMP: instrumentation amplifier; REF: reference; Vout: output voltage.

The four load cells were connected in a bridge configuration and excited by a 9V direct current (DC) power source. As the microforces in the body in response to blood flow (corresponding to the BCG) are very low in magnitude, signal amplification was required. An analog signal-conditioning circuit was developed, which consists of three stages, as shown in Figure 2. The first stage is an alternating current (AC)–coupled instrumentation amplifier (acting as a high-pass filter with *f_c_* of 0.15 Hz) to ensure that the time-varying component (the BCG) from the load cell voltage is enhanced, and the DC component corresponding to the body weight is suppressed. The BCG signal has most of its power in the frequency range of 1-10 Hz [29]; therefore, the second stage is a low-pass filter with *f_c_* of 25 Hz. The third and final stage further amplifies the filtered signal. The circuit has an overall gain of 88 dB and a passband of 0.15-25 Hz.

The filtered and amplified signal output was then converted to digital form using a data acquisition system (National Instruments USB-6351). A digital bandpass filter (0.5-15 Hz) was applied before further processing for heart rate calculation.

### Study Protocol

The physical movement exerted by the person being monitored dominates the signal, leading to the BCG information being unrecognizable. It was hypothesized that a few seconds of relative stillness per minute would be sufficient to obtain a BCG signal and that there would be appropriate windows during typical activities that people do while seated (eg, reading and watching television). To evaluate the prototype efficacy, a study was conducted to emulate real-world activities to determine whether usable BCG data could be extracted from the seat cushion prototype. Five daily life activities were selected: (1) sitting as still as possible, (2) watching a video on a computer screen, (3) reading a magazine, (4) surfing the internet on a computer, and (5) having a conversation with another person.

After obtaining ethical approval from the University of Waterloo Office of Research Ethics (ORE #40503), recruitment for 20 participants aged ≥18 years was determined. Each participant completed a demographic form asking for their age, sex, weight, and height. The participant was then asked to sit on the prototype seat cushion, which was placed on a chair. ECG electrodes were attached, and the ECG recorded to serve as a gold standard comparison for validating BCG data; ECG was captured using a Finapres Medical Systems ECG Module in a Lead-II configuration. Participants were asked to perform each of the five activities for 5 minutes each while BCG and ECG were recorded simultaneously.

### Postprocessing: BCG Data During Activities

Data segments containing identifiable BCG were isolated from segments that were overwhelmed by motion artifacts using a variance-based method. This method was used because the signal voltage undergoes a large variation when there is movement compared with when the participant was sitting still. A moving windowed variance (*Var_mov_*) with a window size of 1 second was computed for the BCG signal, and after trying different thresholds between *mean*(*Var_mov_*) and ¼ *mean*(*Var_mov_*), a threshold value equal to ½ *mean*(*Var_mov_*) was found to be the most appropriate in distinguishing signal segments with motion artifact. All signal segments (windows) with variance above this threshold had too many motion artifacts and were discarded. Of the data that had identifiable BCG, only signal segments with a duration of 5 seconds or longer were kept to ensure that enough consecutive heartbeats were obtained to calculate heartbeats as slow as 40 beats per minute (ie, a bottom threshold that is lower than anyone’s resting heart rate would be).

**Figure 3:**
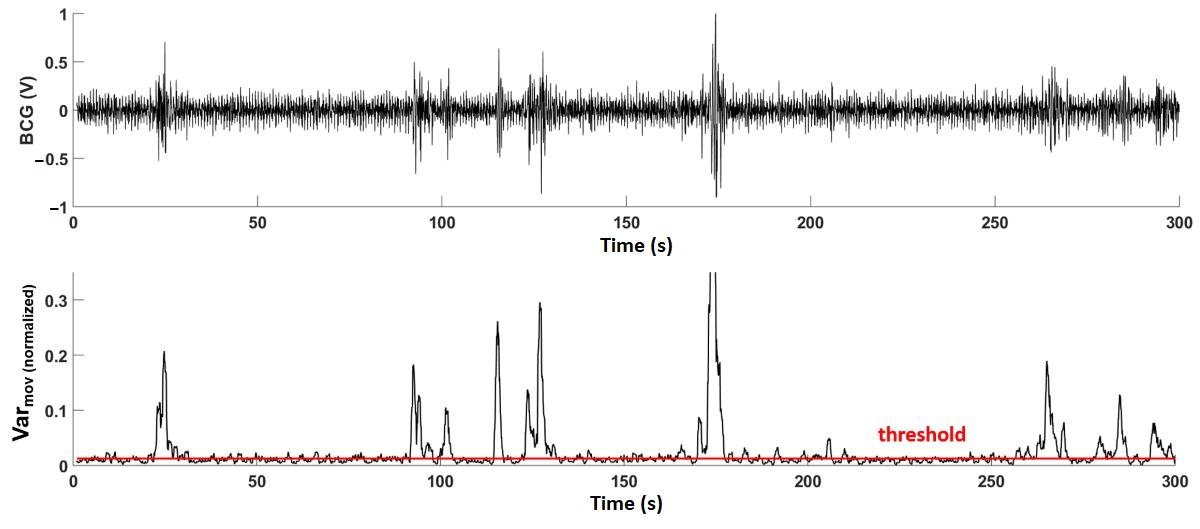
Five minutes of ballistocardiography data obtained from a participant (top). The moving windowed variance function applied to the signal. Signal segments above the threshold were discarded (bottom). BCG: ballistocardiography; Var: variance.

Figure 3 shows 5 minutes of BCG data obtained from a participant during the study and the moving windowed variance applied to the signal. It can be observed that the selected moving variance function is able to detect noisy segments (with motion artifacts) in the BCG data, as they have significantly large variance. This method was applied to BCG recordings of all participants for four activities; the *sitting as still as possible* activity was excluded, as all participants were still during this activity; therefore, most of the data contained a signal that could be directly analyzed.

### Postprocessing: BCG J-Peaks Detection for Heart Rate Calculation

**Figure 4:**
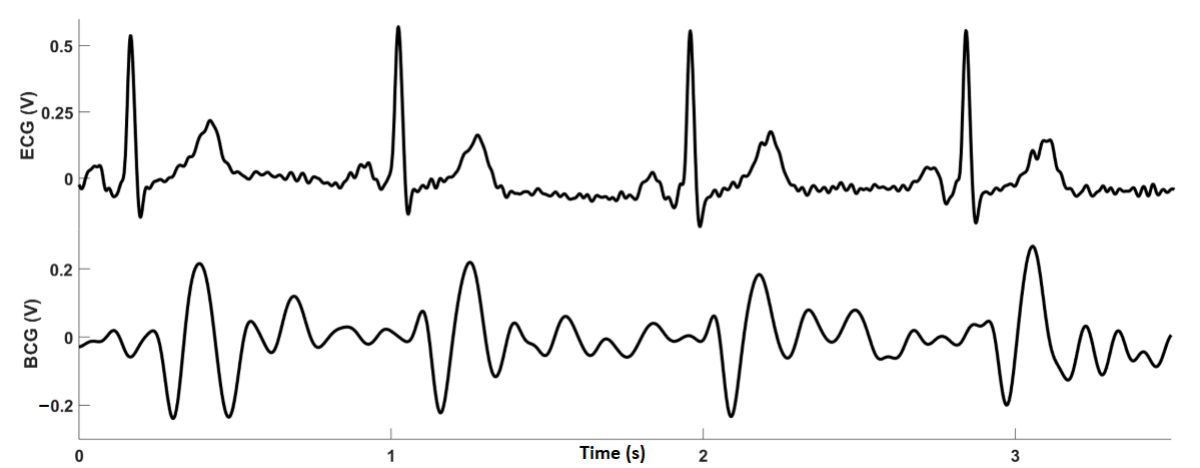
Electrocardiography (top) and ballistocardiography (bottom) recordings obtained simultaneously from a participant. BCG: ballistocardiography; ECG: electrocardiography.

Similar to the R-peaks in an ECG signal, the largest signal amplitude in the BCG during a heartbeat is referred to as the J-peak. A count of these J-peaks can be used to estimate heart rate; however, as J-peaks do not stand out as much from the rest of the signal as R-peaks do in ECG, it can be difficult to detect them (Figure 4). Most J-peak detection methods reported in the literature have extracted heartbeat segments in the BCG signal by using ECG R-peaks as reference [30-32].

#### J-Peak Detection Using Continuous Wavelet Transform

As the aim of this study was to calculate heart rate information solely from the BCG acquired through the seat cushion, methods that do not require ECG had to be considered, such as beat-to-beat heart rate estimation methods [33,34]. An algorithm based on the continuous wavelet transform (CWT) was developed, as wavelet analysis has been performed extensively on heart rate signals [35-38]. CWT is a method that helps in analyzing local variations in frequency in a time series by decomposing the signal into time-frequency space. It provides essential information about the dominant frequencies and how they locate in time. Although the Fourier transform provides accurate information about the frequency content of a signal, it does not provide information about how these frequencies are located in time. The windowed Fourier transform can provide some localized frequency information, but it is not efficient for signals with abrupt changes, such as in the case of BCG [39]. The CWT is an efficient tool in this instance, as it can help identify when (or at what scale of the analyzing wavelet) dominant frequencies are present in the BCG signal. Therefore, the CWT can be used to identify the locations of the heartbeat segments in a BCG signal.

The CWT can be described as follows: Let *x_n_* be a discrete-time signal with a length of *N* (*n*=0, 1, 2, 3, ..., *N*−1), where all *n* points have the same time spacing δt. The CWT of *x_n_*, denoted by *W_n_(s)*, is defined as


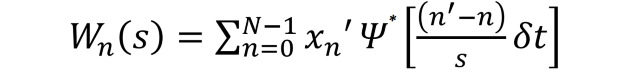


where Ψ^*^ is the complex conjugate of *Ψ(n)*, which is the analyzing wavelet function, and *s* is the scaling factor. The equation mentioned earlier shows that the wavelet transform is obtained by the convolution of *x_n_* with scaled and translated versions of *Ψ(n)* [40], depending on the parameter *s*. The analyzing wavelet *Ψ(n)* has two important properties, that is, it is limited in time and has zero mean [41]. The choice of the analyzing wavelet depends on the analysis being performed; for this case, a Morlet wavelet was used because of its similarity to the BCG waveform and its wide use in biomedical analysis [42-45].

In the methods described in the literature, wavelet transforms have been used for noise cancelation, followed by template matching [35] and the use of different CWT scales for different subjects [36]. However, in this study, the same CWT scale was used for all participants to keep the algorithm autonomous.

**Figure 5:**
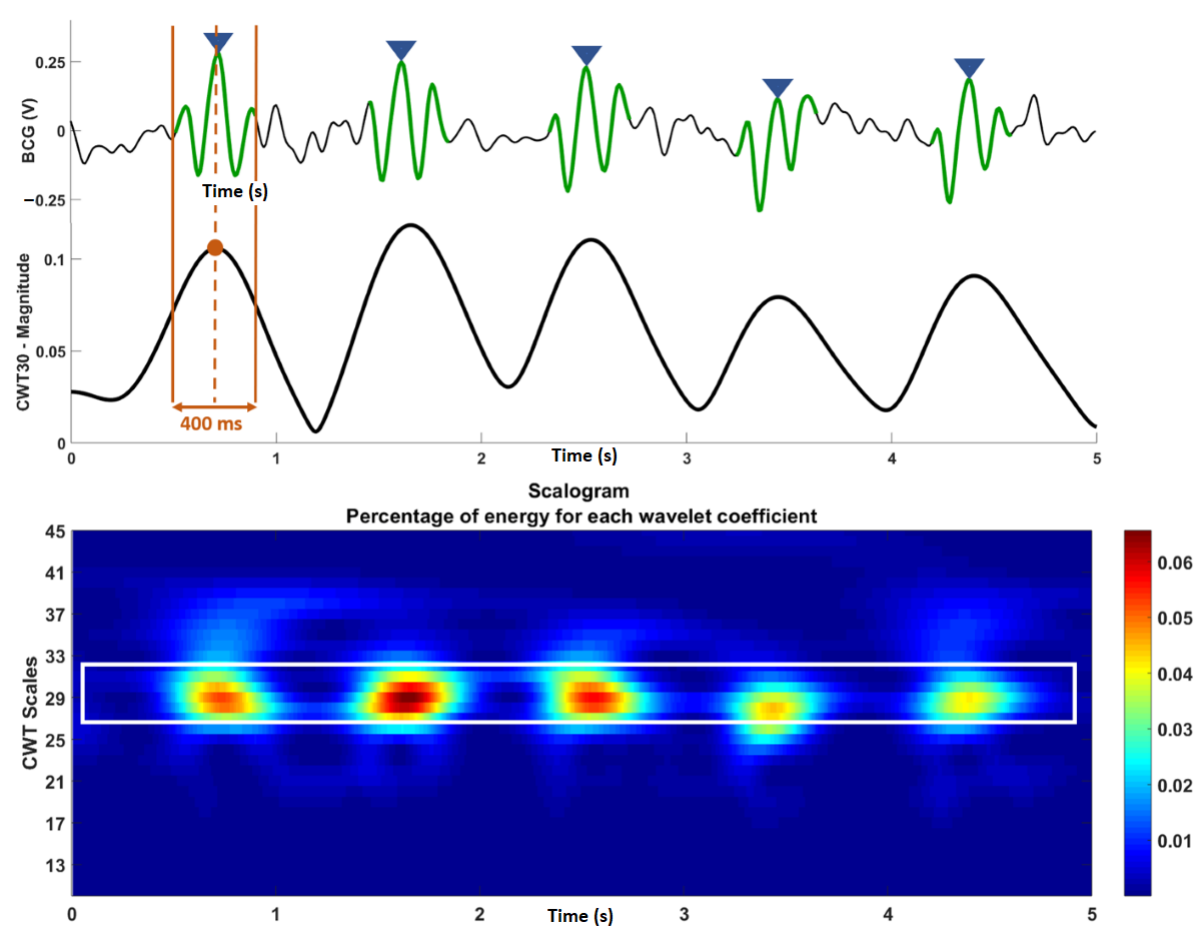
Ballistocardiography (BCG; top) recording with the J-peaks labeled and the continuous wavelet transform (CWT) for the recording with the 400-ms window for heartbeat extraction labeled. Scalogram for CWT of the BCG recording (bottom). BCG: ballistocardiography; CWT: continuous wavelet transform.

Wavelet analysis was performed on the BCG using MATLAB to determine which scales in the CWT provided the most useful information about the time localization of heartbeat segments. A scalogram of the CWT was plotted to observe the scales that contributed the most energy during heartbeat segments. Figure 5 shows a scalogram for a BCG recording obtained during the study, describing the energy for each wavelet coefficient for each scale in time. The figure shows that scales 27-31 provide the most differentiable heartbeat information in the BCG (distinguishable by green, yellow, and red areas in the scalogram image). After testing these five scales, it was observed that scale 30 worked best for all participants, as it provided the largest magnitudes during heartbeat segments. The magnitude plot of the CWT coefficients at scale 30 (*CWT_30_*) is also shown in Figure 5.

It can be observed in Figure 5 that *CWT_30_* has a repetitive pattern with a series of peaks directly related to heartbeat segments in the BCG, indicating that the maximum energy in the BCG lies in the areas around these peaks (in time). Therefore, these peaks of the *CWT*_30_ can help identify BCG heartbeat segments. It was observed that for most participants, the typical BCG waveform was approximately 400 ms in duration; therefore, 400 ms windows (corresponding to heartbeats) from the BCG were extracted using time indices obtained from the locations of the peaks in *CWT_30_*. J-peaks were then autonomously searched for only during these segments, thereby decreasing the chances of incorrectly labeling J-peaks in the BCG. J-peaks were labeled by setting an amplitude threshold equal to the mean of all heartbeat segments. In addition, a time-based threshold was also set, where a J-peak was labeled only if it was at least 500 ms apart from the previous J-peak. This allowed the calculation of heart rates as high as 120 beats per minute, which is well within the normal resting heart rate limit [46].

The CWT analysis and J-peak detection were performed for 1 minute of BCG data obtained from the 60- to 120-second portion of the Sitting Still activity for all 20 participants. This segment was chosen because some participants spent a few seconds adjusting their posture and then remained seated still for the rest of the activity; therefore, data after the first 60 seconds were taken to be representative of the sitting still activity. The performance of the algorithm for J-peak detection was compared with the corresponding R-peaks in the ECG.

#### Estimating Signal-to-Noise Ratio

The signal-to-noise ratio (SNR) for the BCG was estimated using the method presented in a study by Bialasiewicz [44], which was also used in the studies by Inan et al [30], Shao et al [47], and McCall et al [48]. The SNR is estimated using the following equation:


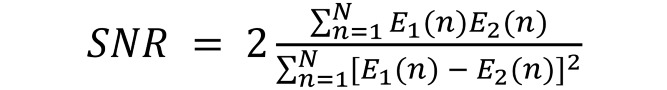


In the abovementioned equation, *E_1_* is the subensemble average of the first 10 seconds of the BCG signal, and *E_2_* is the same for the next 10 seconds. N is the total number of samples in the subensemble average. A subensemble average is the average of all the heartbeat segments in a BCG for a certain duration (in this case, 10 s).

## Results

### Participant Demographics

Table 1 gives an overview of the demographics of the study population.

**Table 1:** Participant demographics (N=20; 13 female and 7 male).

Participant ID	Sex	Age^a^ (years)	Height^b^ (cm)	Weight^c^ (kg)
1	Female	41	171	61
2	Female	23	163	56
3	Male	34	168	59
4	Female	24	160	56
5	Female	23	182	72
6	Male	24	178	65
7	Male	24	183	75
8	Male	24	180	75
9	Female	27	165	56
10	Female	73	160	90
11	Female	22	160	49
12	Male	26	183	100
13	Female	27	158	65
14	Male	29	178	95
15	Male	43	173	128
16	Female	81	168	75
17	Female	75	157	65
18	Female	84	167	63
19	Female	75	178	72
20	Female	80	159	59

^a^Mean 42.9 (SD 24.2).

^b^Mean 169.5 (SD 9.1).

^c^Mean 71.8 (SD 18.8).

### BCG During Activities

**Figure 6:**
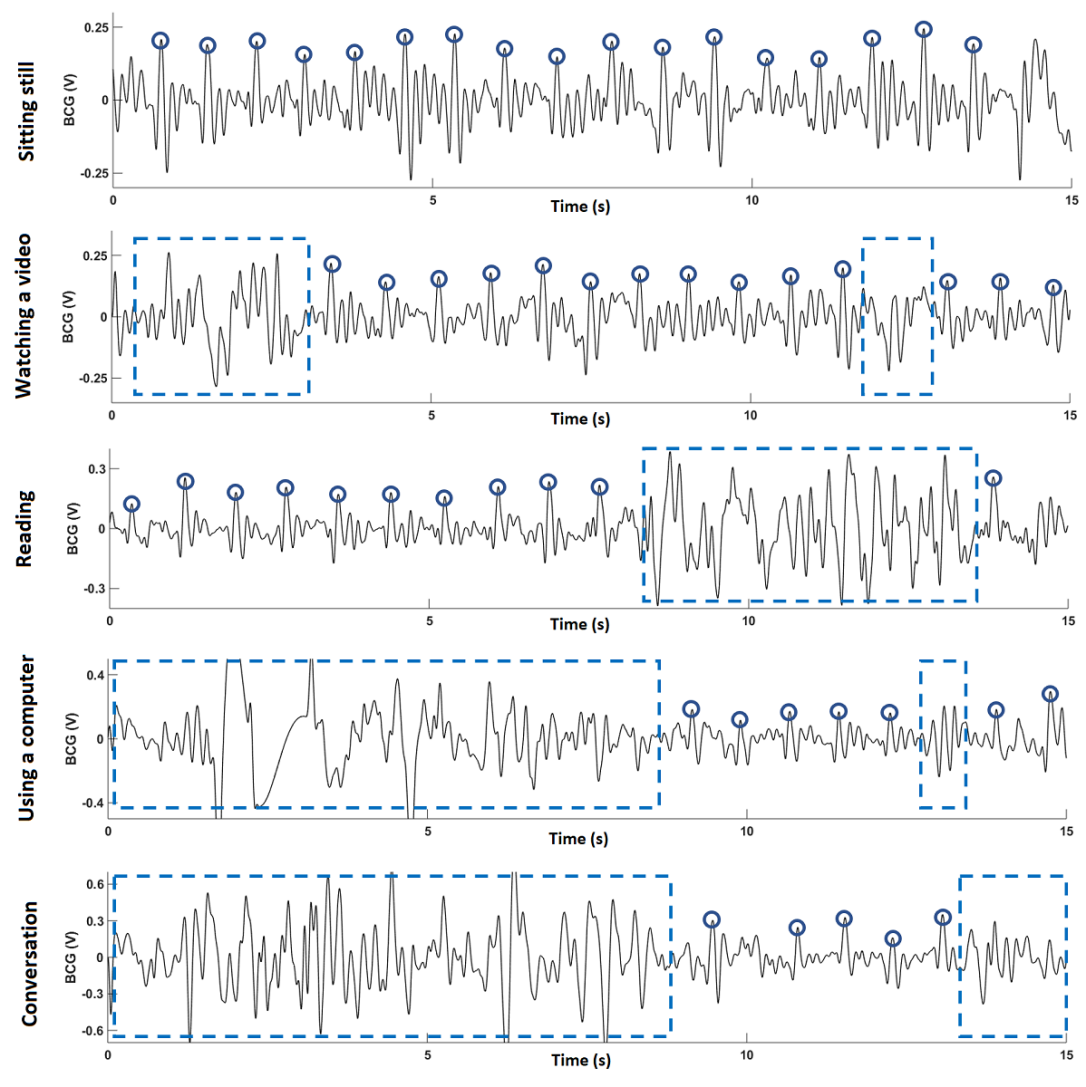
Example data obtained from participant 1 for all five activities. Circles mark J-peaks in the ballistocardiography. Dotted boxes mark noisy signal segments due to motion artifact. BCG: ballistocardiography.

Figure 6 shows 15 seconds of BCG recordings for each of the five activities obtained from participant 1. Table 2 summarizes the results for four simulated activities (the *sitting still* activity was excluded because all participants remained seated still and did not perform any voluntary movement during the recording; therefore, it had long segments of detectable data).

**Table 2:** Results for four simulated activities for 5 minutes each for 20 participants for segments ≥5 seconds.

Activity	Shortest segment (s), mean (SD)	Longest segment (s), mean (SD)	Total number of segments, mean (SD)	Total duration of segments (s), mean (SD)	Total recording containing clean ballistocardiogram data, %
Watching a video	13.1 (13.5)	78.1 (42.4)	9.3 (3.6)	264.6 (24.9)	88.2
Reading	5.9 (1.3)	38.5 (18.6)	12.5 (2.5)	177.9 (50.5)	59.3
Conversation	5.4 (0.4)	20.8 (11.1)	9.8 (3.3)	97.1 (41.3)	32.3
Using a computer	5.4 (0.6)	18.5 (11.2)	9 (3.9)	89.1 (56.1)	29.7

As shown in Figure 6, the J-peaks are readily identifiable when a person is seated still on the prototype. During the watching a video activity, all participants remained seated still for most of the time (an average of 88.2% of the time). For the reading activity, large motion artifact was observed, as turning a page while reading led to significant movement, causing the average duration containing clean BCG to be as low as 59.3%. Similar results were obtained when using a computer activity, as typing on a keyboard leads to significant movement. For the conversation activity, a large variation in time spent sitting still was observed throughout all participants because of different behaviors during a conversation, as some participants used body gestures more often than others. On average, across the four activities, the participants remained seated still for almost one-third of the time.

### CWT-Based J-Peak Detection Method

**Figure 7:**
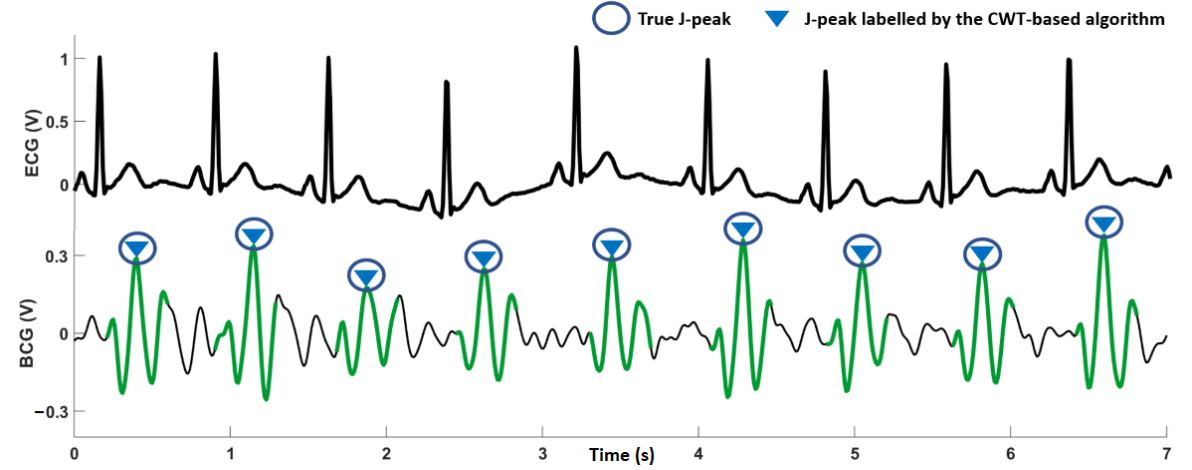
Seven seconds of electrocardiography and ballistocardiography (BCG) recordings for participant 6. The BCG signal is clean, and the algorithm is able to detect all J-peaks correctly. BCG: ballistocardiography; CWT: continuous wavelet transform; ECG: electrocardiography.

**Figure 8:**
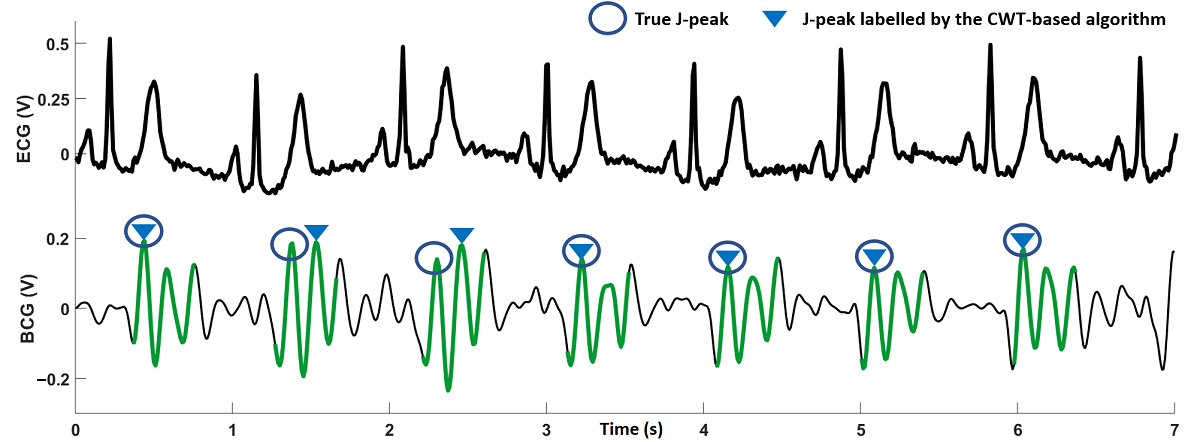
Seven seconds of electrocardiography and ballistocardiography recordings for participant 20. The signal visually appears to be of good quality, but J-peaks amplitudes are not significantly greater than the signal around them, causing the algorithm to label some J-peaks incorrectly. BCG: ballistocardiography; CWT: continuous wavelet transform; ECG: electrocardiography.

**Figure 9:**
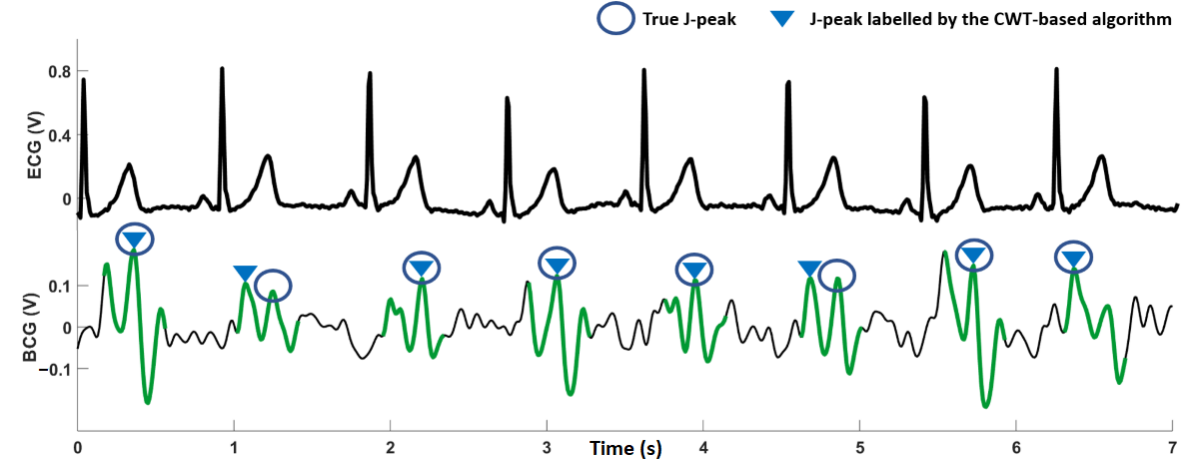
Seven seconds of electrocardiography and ballistocardiography recordings for participant 4. The signal is of poor quality visually, and the J-peaks are not easily distinguishable. This signal also had a very low signal-to-noise ratio. BCG: ballistocardiography; CWT: continuous wavelet transform; ECG: electrocardiography.

The results for the CWT-based J-peak detection algorithm for all 20 participants are summarized in Table 3; the R-peaks in the ECG are included for comparison. A true J-peak positive is a J-peak that was correctly identified by the algorithm. A false positive is a peak that was incorrectly identified as a J-peak. An undetected true J-peak positive is a true J-peak that was not detected (missed) by the algorithm. A visual analysis was conducted to compare the results of the J-peak detection algorithm with the ECG data to establish true positives, false positives, and undetected J-peaks. The sixth column in Table 3 shows the percentage of true J-peak positives compared with the corresponding ECG R-peaks. Overall, the CWT-based algorithm achieved an average accuracy of 91.4% for J-peak detection. The accuracy was more than 90% for 14 participants, whereas for 3 participants (participants 4, 15, and 20), the accuracy was less than 80%. For illustrative purposes, Figure 7, Figure 8, and Figure 9 show 7 seconds of BCG and ECG data from participants 6, 20, and 4, respectively.

**Table 3:** Performance analysis of the continuous wavelet transform–based J-peak detection algorithm.

Participant ID	Total R-peaks	True J-peak positives	False J-peak positives	Undetected true J-peak positives	True J-peak positives^a^, %	Signal-to-noise ratio^b^, dB
1	73	72	0	1	98.6	36.3
2	84	80	2	2	95.2	33.5
3	81	77	1	3	95	43
4	69	46	19	4	66.6	19.9
5	69	61	5	3	88.4	25.7
6	77	77	0	0	100	38.2
7	74	60	8	6	81	26.6
8	67	67	0	0	100	41.2
9	76	73	1	2	96	30.7
10	73	71	1	1	97.2	34.8
11	71	67	3	1	94.3	26.4
12	73	71	1	1	97.2	28.8
13	70	67	1	2	95.7	35.5
14	60	59	0	1	98.3	43.5
15	78	60	16	2	76.9	19.6
16	58	56	2	0	96.5	27.9
17	74	71	3	0	95.9	28.8
18	62	50	11	1	80.6	25.9
19	58	57	1	0	98.2	37.4
20	63	48	15	0	76.1	30.1

^a^Mean 91.4 (SD 9.4).

^b^Mean 31.7 (SD 6.9).

## Discussion

### Principal Findings

The results in Table 2 indicate that there is a substantial number of signal segments (longer than 5 s) during all activities where the person is seated still, resulting in high-quality BCG data. The presence of a large number of these clean heartbeat segments enables the extraction of heartbeat data, which suggests that the seat cushion can be an effective method for continuous monitoring of heart rate using BCG.

Visual analysis of the data in Table 1 was performed for possible correlations between BCG signal shape and age, sex, or weight; none were found.

The CWT-based algorithm performed well for the participants in this study. For example, for participant 6, the CWT-based method was able to correctly identify all J-peaks in the BCG trace, as shown in Figure 7. The amplitudes of the J-peaks are larger than the signal segments around them, which is also evident from the high SNR obtained for the BCG signal (Table 3). The BCG for participant 20 (Figure 8) shows that the signal visually appears to be of good quality, with an SNR just below average. However, the amplitudes of the J-peaks for this participant were not much larger than the other peaks in the signal around them. This caused the algorithm to label some J-peaks incorrectly and correctly identify only 76% (48/63) of true J-peak positives. As shown in Figure 9 for participant 4, it can be observed that the signal quality is poor; the J-peaks are not clearly discernible because they have low amplitudes compared with the signal around them during a heartbeat. This is corroborated by the low SNR obtained for this signal (19.9 dB). The accuracy of J-peak detection was the lowest for this participant. For participant 20, as mentioned earlier, a low J-peak detection accuracy was obtained for a relatively high SNR. This is a limitation of the algorithm, as it can generate inaccurate values of heart rate because of incorrect identification of J-peaks, even for BCG signals that are visually robust.

Excluding the three outliers, the algorithm resulted in an average accuracy of 94.66% (1136/1200). Commercially available wearable devices that use PPG as the signal to calculate heart rate have been evaluated in studies for accuracies between 79.8% to 99.1% [49] and 94.04% to 94.14% [50]. This suggests that the performance of the seat cushion prototype is comparable with that of commercially available wearable devices.

We note that the detection accuracy for the proposed algorithm would be increased by using ECG R-peaks as a reference to detect J-peaks. However, this research focused on calculating heart rate by having a person simply seated on a cushion without them having to wear or attach any sensors, which is a requirement for the acquisition of an ECG signal.

The limitations of the J-peak detection algorithm can be improved. In this study, the CWT scales were used to highlight heartbeat segments to detect J-peaks; however, it would be worthwhile to investigate whether a scale of the CWT can directly provide heart rate information, as it has a repetitive nature similar to that of the BCG (Figure 5). This would increase the algorithm speed while decreasing the computational resources. Machine learning–based approaches are another area worth exploring to detect patterns in BCG across various BCG signals and thus further improve accuracy.

### Conclusions

This paper presents research on creating a seat cushion for ambient heart rate monitoring using BCG. The seat cushion was developed using off-the-shelf components and resulted in a cost-effective prototype that performed robust BCG detection. The CWT-based algorithm we developed for autonomous J-peak detection achieved 94.6% accuracy (excluding three outliers), making it a viable alternative to existing health monitoring technologies. The solution presented here is portable, unobtrusive, and can be easily integrated into a living environment for zero-effort heart rate monitoring.

Emerging research that captures ECG without requiring electrodes attached to the skin, such as coupled capacitance, could be explored to improve the robustness of detecting heart rate as well as potentially supporting measurement of other cardiac information, such as blood pressure. The system input–referred noise can be calculated to quantify noise and identify changes in the seat cushion design that could lead to cleaner BCG signals. To better exclude BCG segments involving significant physical movement, a sensor fusion approach could be explored by sensing acceleration using an accelerometer to detect this movement in real time. Developments such as these will shape the future of unobtrusive and more pervasive heart rate monitoring.

## Abbreviations

AAL: ambient assisted living
BCG: ballistocardiography
CWT: continuous wavelet transform
DC: direct current
ECG: electrocardiography
PPG: photoplethysmography
SNR: signal-to-noise ratio
ZET: zero-effort technology

## References

[1] (2017). Cardiovascular diseases (CVDs). World Health Organization.

[2] (2017). Heart disease in Canada. Public Health Agency of Canada, Government of Canada.

[3] (2017). Cardiovascular disease cost will exceed $1 trillion by 2035: nearly half of Americans will develop pre-existing cardiovascular disease conditions, analysis shows. RTI International.

[4] United Nations Department of Economic and Social Affairs (2020). World Population Ageing 2019.

[5] World Health Organization (2008). The Global Burden of Disease: 2004 update.

[6] Gaugler JE, Duval S, Anderson KA, Kane RL (2007). Predicting nursing home admission in the U.S: a meta-analysis. BMC Geriatrics.

[7] Konstam MA (2012). Home monitoring should be the central element in an effective program of heart failure disease management. Circulation.

[8] Khan Y, Ostfeld AE, Lochner CM, Pierre A, Arias AC (2016). Monitoring of vital signs with flexible and wearable medical devices. Adv Mater.

[9] Chen M, Ma Y, Song J, Lai C, Hu B (2016). Smart clothing: connecting human with clouds and big data for sustainable health monitoring. Mobile Netw Appl.

[10] Bonato P (2010). Advances in wearable technology and its medical applications. Proceedings of the Annual International Conference of the IEEE Engineering in Medicine and Biology.

[11] Finni T, Hu M, Kettunen P, Vilavuo T, Cheng S (2007). Measurement of EMG activity with textile electrodes embedded into clothing. Physiol Meas.

[12] Zhang F, Yu Y, Zhong J (2019). Research status and development prospects of human vital signs monitoring clothing. IOP Conf Ser: Earth Environ Sci.

[13] Koyama Y, Nishiyama M, Watanabe K (2018). Smart textile using hetero-core optical fiber for heartbeat and respiration monitoring. IEEE Sensors J.

[14] Boger J, Young V, Hoey J, Jiancaro T, Mihailidis A (2018). Zero-effort technologies: considerations, challenges and use in health, wellness, and rehabilitation. 2nd ed. Synthesis Lectures on Assistive, Rehabilitative, and Health-Preserving Technologies.

[15] Gordon JW (1877). Certain molar movements of the human body produced by the circulation of the blood. J Anat Physiol.

[16] Starr I, Rawson AJ, Schroeder HA, Joseph NR (1939). Studies on the estimation of cardiac ouptut in man, and of abnormalities in cardiac function, from the heart's recoil and the blood's impacts; the ballistocardiogram. Am J Physiol-Legacy Content.

[17] Shin JH, Lee KM, Park KS (2009). Non-constrained monitoring of systolic blood pressure on a weighing scale. Physiol Meas.

[18] Ashouri H, Orlandic L, Inan O (2016). Unobtrusive estimation of cardiac contractility and stroke volume changes using ballistocardiogram measurements on a high bandwidth force plate. Sensors (Basel).

[19] Chang IS, Javaid A, Boger J, Arcelus A, Mihailidis A (2018). Design and evaluation of an instrumented floor tile for measuring older adults’ cardiac function at home. Gerontechnology.

[20] Javaid AQ, Wiens AD, Fesmire NF, Weitnauer MA, Inan OT (2015). Quantifying and reducing posture-dependent distortion in ballistocardiogram measurements. IEEE J Biomed Health Inform.

[21] He D, Winokur E, Sodini C (2011). A continuous, wearable, and wireless heart monitor using head Ballistocardiogram (BCG) and head electrocardiogram (ECG). Proceedings of the Annual International Conference of the IEEE Engineering in Medicine and Biology Society.

[22] Deliere Q, Migeotte P, Neyt X, Funtova I, Baevsky RM, Tank J, Pattyn N (2013). Cardiovascular changes in parabolic flights assessed by Ballistocardiography. Proceedings of the 35th Annual International Conference of the IEEE Engineering in Medicine and Biology Society (EMBC).

[23] Paajanen M, Lekkala J, Kirjavainen K (2000). ElectroMechanical Film (EMFi) — a new multipurpose electret material. Sens Actuator A Phys.

[24] Baek HJ, Chung GS, Kim LL, Park KS (2012). A smart health monitoring chair for nonintrusive measurement of biological signals. IEEE Trans Inform Technol Biomed.

[25] Walter M, Eilebrecht B, Wartzek T, Leonhardt S (2011). The smart car seat: personalized monitoring of vital signs in automotive applications. Pers Ubiquit Comput.

[26] Pinheiro E, Postolache O, Girão P (2012). Study on ballistocardiogram acquisition in a moving wheelchair with embedded sensors. Metrol Measure Sys.

[27] Karki S, Lekkala J (2008). Film-type transducer materials PVDF and EMFi in the measurement of heart and respiration rates. Proceedings of the 30th Annual International Conference of the IEEE Engineering in Medicine and Biology Society.

[28] Conn NJ, Schwarz KQ, Borkholder DA (2019). In-home cardiovascular monitoring system for heart failure: comparative study. JMIR Mhealth Uhealth.

[29] Gomez-Clapers J, Serra-Rocamora A, Casanella R, Pallas-Areny R (2014). Towards the standardization of ballistocardiography systems for J-peak timing measurement. Measurement.

[30] Inan OT, Etemadi M, Wiard RM, Giovangrandi L, Kovacs GT (2009). Robust ballistocardiogram acquisition for home monitoring. Physiol Meas.

[31] Wiens A, Etemadi M, Klein L, Roy S, Inan O (2014). Wearable ballistocardiography: preliminary methods for mapping surface vibration measurements to whole body forces. Proceedings of the 36th Annual International Conference of the IEEE Engineering in Medicine and Biology Society.

[32] Kim CS, Carek AM, Inan OT, Mukkamala R, Hahn J (2018). Ballistocardiogram-based approach to cuffless blood pressure monitoring: proof of concept and potential challenges. IEEE Trans Biomed Eng.

[33] Bruser C, Stadlthanner K, de Waele S, Leonhardt S (2011). Adaptive beat-to-beat heart rate estimation in ballistocardiograms. IEEE Trans Inform Technol Biomed.

[34] Lydon K, Su B, Rosales L, Moein E, Ho KC, Rantz M, Skubic M (2015). Robust heartbeat detection from in-home ballistocardiogram signals of older adults using a bed sensor. Proceedings of the 37th Annual International Conference of the IEEE Engineering in Medicine and Biology Society (EMBC).

[35] Postolache O, Silva GP, Postolache G, Pereira M (2007). Vital signs monitoring system based on EMFi sensors and wavelet analysis. Proceedings of the IEEE Instrumentation & Measurement Technology Conference IMTC 2007.

[36] Noh Y, Kew H, Jeong D (2009). BGG monitoring system using unconstrained method with daubechies wavelet transform. Proceedings of the 5th International Conference on Intelligent Manufacturing & Logistics System.

[37] Giaberte S, Gomez-Clapers J, Cassanella R, Pallas-Areny R (2010). Heart and respiratory rate detection on a bathroom scale based on the ballistocardiogram and the continuous wavelet transform. Proceedings of the Annual International Conference of the IEEE Engineering in Medicine and Biology.

[38] Alvarado C, Arregui J, Ramos J, Pallas-Areny R (2005). Automatic detection of ECG ventricular activity waves using continuous spline wavelet transform. Proceedings of the 2nd International Conference on Electrical and Electronics Engineering.

[39] Kaiser G (2011). A friendly guide to wavelets. Modern Birkhäuser Classics.

[40] Torrence C, Compo GP (1998). A practical guide to wavelet analysis. Bull Amer Meteor Soc.

[41] Farge M (1992). Wavelet transforms and their applications to turbulence. Annu Rev Fluid Mech.

[42] Leandro JJ, Cesar J, Jelinek HF (2001). Blood vessels segmentation in retina: preliminary assessment of the mathematical morphology and of the wavelet transform techniques. Proceedings XIV Brazilian Symposium on Computer Graphics and Image Processing.

[43] Goelz H, Jones RD, Bones PJ (2000). Wavelet analysis of transient biomedical signals and its application to detection of epileptiform activity in the EEG. Clin Electroencephalogr.

[44] Bialasiewicz JT (2015). Application of wavelet scalogram and coscalogram for analysis of biomedical signals. Proceedings of the World Congress on Electrical Engineering and Computer Systems and Science (EECSS 2015).

[45] Mgdob H, Torry J, Al-Naami B (2003). Application of Morlet transform wavelet in the detection of paradoxical splitting of the second heart sound. Proceedings in Computers in Cardiology 2003.

[46] Jose AD, Collison D (1970). The normal range and determinants of the intrinsic heart rate in man. Cardiovasc Research.

[47] Shao D, Tsow F, Liu C, Yang Y, Tao N (2017). Simultaneous monitoring of ballistocardiogram and photoplethysmogram using a camera. IEEE Trans Biomed Eng.

[48] McCall C, Stuart Z, Wiard R, Inan O, Giovangrandi L, Cuttinto C, Kovacs G (2014). Standing ballistocardiography measurements in micogravity. Proceedings of the 36th Annual International Conference of the IEEE Engineering in Medicine and Biology Society.

[49] El-Amrawy F, Nounou MI (2015). Are currently available wearable devices for activity tracking and heart rate monitoring accurate, precise, and medically beneficial?. Healthc Inform Res.

[50] Nelson BW, Allen NB (2019). Accuracy of consumer wearable heart rate measurement during an ecologically valid 24-hour period: intraindividual validation study. JMIR Mhealth Uhealth.

